# Influenza A(H7N9) Virus Transmission between Finches and Poultry

**DOI:** 10.3201/eid2104.141703

**Published:** 2015-04

**Authors:** Jeremy C. Jones, Stephanie Sonnberg, Richard J. Webby, Robert G. Webster

**Affiliations:** St. Jude Children’s Research Hospital, Memphis, Tennessee, USA

**Keywords:** influenza, influenza virus, influenza A(H7N9), subtype H7N9, low pathogenicity avian influenza, passerine birds, finches, poultry, interspecies transmission, virus transmission, quail, chicken, waterborne, water, viruses, contact, transmission model

## Abstract

Low pathogenicity avian influenza A(H7N9) virus has been detected in poultry since 2013, and the virus has caused >450 infections in humans. The mode of subtype H7N9 virus transmission between avian species remains largely unknown, but various wild birds have been implicated as a source of transmission. H7N9 virus was recently detected in a wild sparrow in Shanghai, China, and passerine birds, such as finches, which share space and resources with wild migratory birds, poultry, and humans, can be productively infected with the virus. We demonstrate that interspecies transmission of H7N9 virus occurs readily between society finches and bobwhite quail but only sporadically between finches and chickens. Inoculated finches are better able to infect naive poultry than the reverse. Transmission occurs through shared water but not through the airborne route. It is therefore conceivable that passerine birds may serve as vectors for dissemination of H7N9 virus to domestic poultry.

## Abstract 2

Transmission via shared water implicates passerine birds as possible vectors for dissemination of this virus.

In spring 2013, novel avian influenza A(H7N9) viruses emerged in eastern China (*1*). These viruses are reassortants of subtype H7 and H9N2 viruses from wild birds and poultry (*2*,*3*) and were detected in humans and subsequently in chickens, ducks, pigeons, water, and soil at bird markets (*4*,*5*). H7N9 virus does not induce clinical signs in poultry (*6*), and genetic analyses show a monobasic cleavage site in the hemagglutinin (HA) protein (*1*); H7N9 virus is therefore classified as a low pathogenicity avian influenza virus (LPAIV). However, the virus can infect humans and cause severe disease (*7*). Human infection with H7N9 virus was first reported in China in March 2013 (*8*). By October 2, 2014, a total of 453 confirmed cases and 175 associated deaths had been reported (http://www.who.int/influenza/human_animal_interface/influenza_h7n9/riskassessment_h7n9_2Oct14.pdf?ua=1). Despite their avian genetic background, some H7N9 viruses have HA and polymerase protein mutations that confer a replication advantage in mammals (*1*). Human infection has been associated with exposure to poultry or live poultry markets (*7*,*9*); market closings likely contributed to infection declines in mid-2013 (*10*). Nevertheless, H7N9 virus persists in poultry, and human infections surged in the late 2013, demonstrating that this virus is an ongoing public health threat (*11*).

The polymerase acidic (PA) and polymerase basic 2 genes derived from A/Anhui/1/2013 (H7N9)–like virus are homologous to those from A/brambling/Beijing/16/2012 (H9N2) (*1*,*8*), a strain isolated from a brambling (*Fringilla montifringilla*, a small passerine bird). In addition, during surveillance in 2013, the influenza strain A/tree sparrow/Shanghai/01/2013 (H7N9) was identified in a tree sparrow (*Passer montanus,* a passerine bird) found at a site where migratory and local birds congregate (*12*). 

We previously showed that society finches (*Lonchura striata domestica*), zebra finches (*Taeniopygia guttata*), sparrows (*P. domesticus*), and parakeets (*Melopsittacus undulates*) are susceptible to H7N9 virus and shed virus into water (*13*). The birds used in those experiments are examples of passerine and psittacine birds, which include individual species that are migratory, peridomestic, and domesticated. The interaction of wild birds, humans, and domesticated animals may contribute to the maintenance and spread of H7N9 virus. To further address the contribution of passerines to the ecology of H7N9 virus, we modeled potential interspecies virus transmission by using society finches (a passerine bird) and poultry (bobwhite quail and chickens) and determined the route of virus transmission.

## Methods

### Viruses and Facilities

For the experiments, we used A/Anhui/1/2013 (H7N9) (hereafter referred to as Anhui/1) from an index human patient (*14**–**18*) and a poultry isolate, A/chicken/Rizhao/867/2013 (H7N9) (hereafter referred to as Ck/Rizhao), from an original swab sample. Anhui/1 and Ck/Rizhao (provided by Huachen Zhu [Shantou University, Shantou, China] and Yi Guan [University of Hong Kong, Hong Kong, China]) were propagated and titrated in embryonated chicken eggs (*13*). Pooled allantoic fluid was used as virus stock, and the viruses were passaged 3 times in eggs. The genomic sequence of the Anhui/1 sample corresponded to those of an isolate from GISAID (Global Initiative on Sharing Avian Influenza Data; accession no. EPI_ISL_138739), and genomic sequences of the Ck/Rizhao sample corresponded to those of an isolate from GenBank (accession nos. KF260954, KF259043, and KF259731). Experiments were performed under Animal Biosafety Level 3+ conditions as defined in US Department of Agriculture regulatory documents 9 CFR part 121 and 7 CFR part 331 (http://www.aphis.usda.gov/programs/ag_selectagent/downloads/FinalRule3-18-05.pdf).

### Animals

Study birds were of both sexes and included 3- to 6-month-old society finches (*L. striata domestica*) (Birds Express, South El Monte, CA, USA); 5-week-old white leghorn hens (*Gallus gallus domesticus*) (McMurray Hatchery, Webster City, IA, USA); and 16-week-old bobwhite quail (*Colinus virginianus*) (B&D Game Farm, Harrah, OK, USA). The birds were quarantined for 1 week, and prechallenge swab samples were confirmed influenza virus–negative by egg isolation. Food and water were provided ad libitum. Animal experiments were approved by the St. Jude Children’s Research Hospital Animal Care and Use Committee and complied with all applicable US regulations.

### Inoculation and Sampling

Birds were inoculated intranasally, intraocularly, or orally with 10^5^ log_10_ 50% egg infectious doses (EID_50_) of virus in 100 μL (finches) or 500 μL (poultry) of phosphate buffered saline. Oropharyngeal and cloacal swab samples were collected on days postinoculation (dpi) 2, 4, 6, 8, 10, and 13. Water samples (500 μL) were obtained 1–4 and 8 dpi. Samples were titrated in eggs (*13*).

### Interspecies Transmission Study Design

**Figure 1:**
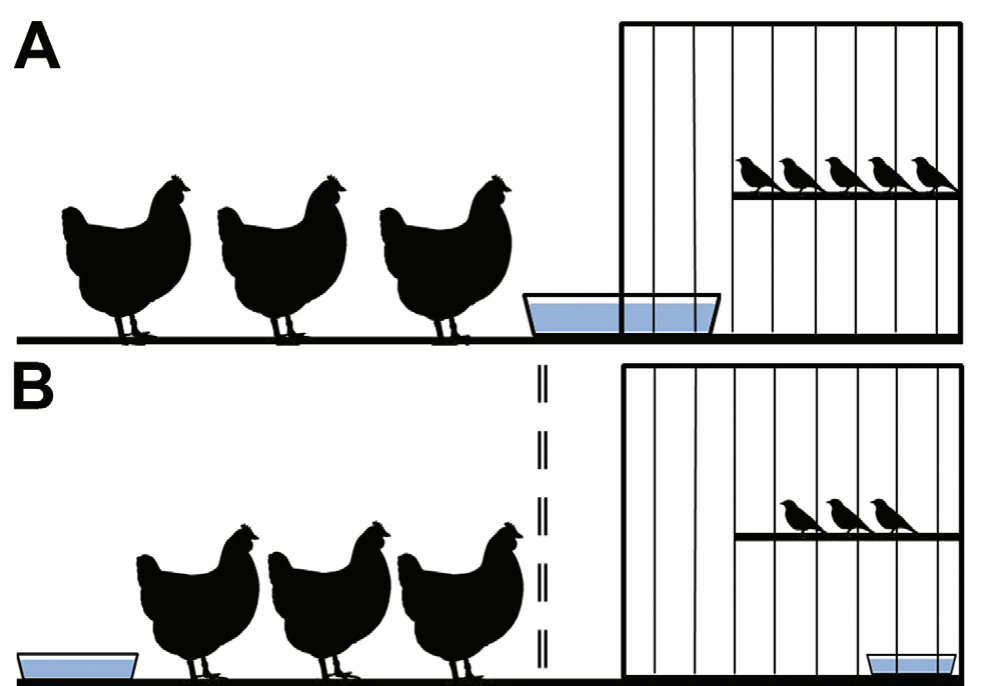
Design model for an interspecies study of influenza A(H7N9) virus transmission. Birds were housed in a cage-within-a-cage setup with a 30 cm × 41 cm × 41 cm finch cage placed within a 97 cm × 58 cm × 53 cm poultry cage. A) Waterborne transmission was examined by sliding a 15 cm × 25 cm pan containing ≈1 L water halfway into a notched hole in the finch cage. All birds had shared access to the water, but poultry and finches were excluded from physical contact with each other. B) Airborne transmission was examined by inserting an air-permeable cage barrier (represented by double-dashed line) between the poultry and the finch cage and providing separate water supplies so that poultry and finches had no direct physical contact and did not share food or water resources.

Birds were cohoused in a cage-within-a-cage setup. Poultry (n = 3) were housed in a 97 cm × 58 cm × 53 cm cage that contained a 30 cm × 41 cm × 41 cm cage housing finches (n = 4 or 5). This setup was used in duplicate for each experiment, and the data obtained from each set of cages were combined. Waterborne transmission was examined by sliding a water pan (15 cm × 25 cm) halfway into a notched hole in the finch cage (Figure 1, panel A); birds shared water but did not have physical contact. For airborne transmission experiments, an air-permeable barrier separated poultry from the finch cage, and water sources were separate (Figure 1, panel B). Each day, 1 L of filtered, nonchlorinated water was provided by topping off the supply remaining in the water pans; every 96 h, the full water supply in the pans was replaced.

### Necropsy

Necropsies were performed on birds that died during the study. Trachea and/or lung and intestine samples were harvested (Table 1) and homogenized in 1 mL of saline–antimicrobial drugs. Virus was isolated and titrated in eggs (*13*).

**Table 1:** Virus isolation from organs of dead birds in an interspecies study of influenza A(H7N9) virus transmission*

Bird species	Time of death, dpi	Influenza virus exposure	Transmission			Virus titer, log_10_ EID_50_/mL†	
			Route	Direction		Trachea and/or lung	Intestine
Naive contact							
Finch	4	A/Anhui/1/2013 (H7N9)	Airborne	Chicken → Finch		0	0
Finch	5	A/Anhui/1/2013 (H7N9)	Waterborne	Chicken → Finch		4.3 (combined)	0
Finch	5	A/chicken/Rizhao/867/2013 (H7N9)	Waterborne	Chicken → Finch		6.5 (combined)	0
Quail	15	A/Anhui/1/2013 (H7N9)	Waterborne	Finch → Quail		4.7 (trachea); 5.5 (lung)	ND
Quail	15	A/Anhui/1/2013 (H7N9)	Waterborne	Finch → Quail		7.3 (trachea); 8.3 (lung)	ND
Quail	10	A/chicken/Rizhao/867/2013 (H7N9)	Waterborne	Finch → Quail		6.5 (trachea); 7.5 (lung)	ND
Inoculated							
Finch	6	A/Anhui/1/2013 (H7N9)	Waterborne	Finch → Chicken		4.5 (combined)	0
Finch	2	A/Anhui/1/2013 (H7N9)	Waterborne	Finch → Quail		2.5 (trachea); 2.3 (lung)	ND
Quail	15	A/Anhui/1/2013 (H7N9)	Airborne	Quail → Finch		3.3 (trachea); 4.5 (lung)	ND

*dpi, days postinfection; EID_50_, 50% egg infectious dose; ND, not determined. †Assessed in embryonated chicken eggs.

### Serologic Testing

Before beginning the experiments, we tested >5 birds from each species for influenza A virus antibodies; all results were negative. On dpi 16, we collected blood samples from the surviving birds and tested them for H7N9 virus seroconversion by using the IDEXX AI MultiS-Screen Ab Test (IDEXX Laboratories, Westbrook, ME, USA) according to the manufacturer’s instructions.

### Statistical Analyses

Mean infectious titers were compared by using the 1-tailed Student *t*-test in Excel (Microsoft, Redmond, WA, USA) or GraphPad Prism v5 (La Jolla, CA, USA). Area under the curve (AUC) analysis for cumulative shedding was performed by using GraphPad Prism v5.

## Results

### Waterborne Transmission between Society Finches and Chickens

Waterborne virus transmission between finches and chickens was investigated by inoculating 1 species (donors) with 10^5^ log_10_ EID_50_ units of virus and pairing the donor birds with the naive bird species (water contacts) (Figure 1, panel A). We previously observed little to no shedding in society finches via the cloaca (*13*); thus, in this study, we collected swab samples at a single time point (4 dpi). We obtained oropharyngeal and cloacal swab samples from poultry at each time point.

**Figure 2:**
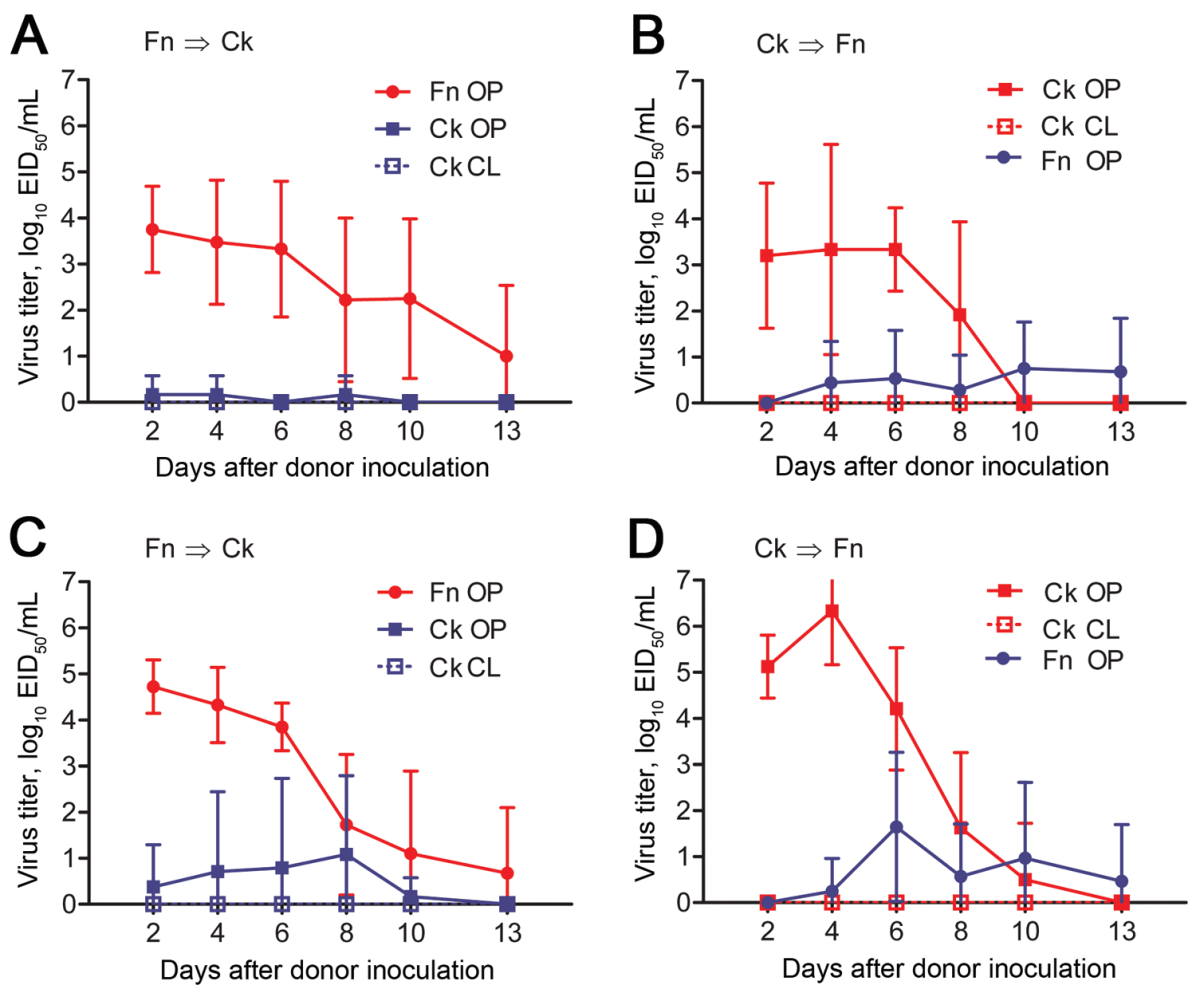
Waterborne transmission of virus between chicken and finches in an interspecies study of influenza A(H7N9) virus transmission. Finches (n = 8 or 10) and chickens (n = 6) were inoculated with strain A/Anhui/1/2013 (H7N9) (A, B) or A/chicken/Rizhao/867/2013 (H7N9) (C, D) and paired with naive birds in an environment in which physical contact was prevented but water was shared (Figure 1, panel A). Swab samples were obtained from birds every 48 h, and virus titers were determined in embryonated chicken eggs. Data are the average titer per time point ± SD. Directionality of transmission (i.e., infected → naive) is indicated in the top left of each panel. Red indicates infected animals; blue indicates naive animals. Ck, chicken; CL, cloacal swab sample; EID_50_, 50% egg infectious dose; Fn, finch; OP, oropharyngeal swab sample.

All donor finches were productively infected with Anhui/1 or Ck/Rizhao and shed virus by the oropharyngeal route for 10–13 dpi (Figure 2, panels A, C; Table 2). No virus was detected in cloacal swab samples. Using AUC analysis, including all animals, we found no statistical difference between cumulative shedding of the 2 viruses from donor finches.

**Table 2:** Virus transmission, shedding, sickness, and death among birds in an interspecies study of influenza A(H7N9) virus transmission*

Virus, donor/naive contact, transmission route	No. with OP virus shedding/no. total (%)			Clinical signs of illness†		No. died/no. total (%) species
	Donors	Naive contacts		Donors	Naive contacts	
A/Anhui/1/2013 (H7N9)						
Finch/chicken						
Waterborne	10/10 (100)	0/6 (0)		−	−	1/10 (10) finches
Airborne	2/2 (100)	0/3 (0)		−	−	0
Finch/quail						
Waterborne	10/10 (100)	6/6 (100)		−	+	1/10 (10) finches; 2/6 (33) quail
Airborne	3/3 (100)	0/3 (0)		−	−	0
Chicken/finch						
Waterborne	6/6 (100)	3/8 (38)		−	−	1/8 (13) finch
Airborne	3/3 (100)	0/2 (0)		−	−	1/2 (50) finch
Quail/finch						
Waterborne	6/6 (100)	2/10 (20)		+	−	0
Airborne	3/3 (100)	0/3 (0)		+	−	1/3 (33) quail
A/chicken/Rizhao/867/2013 (H7N9)						
Finch/chicken						
Waterborne	10/10 (100)	2/6 (33)		−	−	0
Airborne	NA	NA		NA	NA	NA
Finch/quail						
Waterborne	10/10 (100)	6/6 (100)		−	−	1/6 (17) quail
Airborne	NA	NA		NA	NA	NA
Chicken/finch						
Waterborne	6/6 (100)	4/8 (50)		−	−	1/8 (13) finch
Airborne	NA	NA		NA	NA	NA
Quail/finch						
Waterborne	6/6 (100)	2/10 (20)		−	−	0
Airborne	NA	NA		NA	NA	NA

*OP, oropharyngeal route; NA, not applicable; +, symptoms present; −, symptoms not present. †Clinical signs or symptoms were observed at least once and included >1 of the following: hunched posture, ruffled feathers, and lethargy.

During oropharyngeal sampling of the naive water contacts, we considered the possibility that we were obtaining transient virus that the birds acquired during recent drinking. To differentiate transiently acquired virus from replicated/shed virus, we defined a transmission event as an instance when samples from a naive water contact contained >2.5 log_10_ EID_50_/mL of virus and/or when the bird shed during >2 consecutive time points. Under such criteria, waterborne transmission from finches to chickens was limited. Of 6 water-contact chickens paired with Anhui/1-donor finches, 2 shed <2 logs of virus for a single time point, which did not meet our transmission criteria (Figure 2, panel A; Table 2). Two water-contact chickens paired with Ck/Rizhao-donor finches shed virus over multiple time points (Figure 2, panel C; Table 2). Water-contact chickens shed virus by the oropharyngeal route; virus was not detected in cloacal swab samples. Cumulative shedding was not significantly different for the 2 viruses in the water contacts.

In the converse experiment, all donor chickens were productively infected with both viruses and shed virus an average of 10 days (Figure 2, panels B, D; Table 2). Chickens shed virus by the oropharyngeal route only, and cumulative shedding was statistically higher in birds inoculated with Ck/Rizhao than with Anhui/1 (AUC analysis, p<0.01).

Three water-contact finches paired with Anhui/1-donor chickens met our transmission criteria, although average virus titers were low (peak titers 1.0–2.8 log_10_ EID_50_/mL) (Figure 2, panel B; Table 2). Four water-contact finches paired with Ck/Rizhao-donor chickens became infected; 3 had low virus titers (peak titer 1.0–3.5 log_10_ EID_50_/mL), but the fourth finch had higher virus titers (peak titer 4.3 log_10_ EID_50_/mL) and shed virus for 7 days (4 sampling time points) (Figure 2, panel D; Table 2). All water-contact finches shed virus by the oropharyngeal route; no virus was isolated from cloacal swab samples. Cumulative shedding of the 2 viruses did not differ statistically in the water-contact finches. Overall, our data showed that, when water resources are shared, virus transmission between society finches and chickens is sporadic, and contact birds generally shed virus at low titers for short periods.

### Waterborne Transmission between Finches and Quail

**Figure 3:**
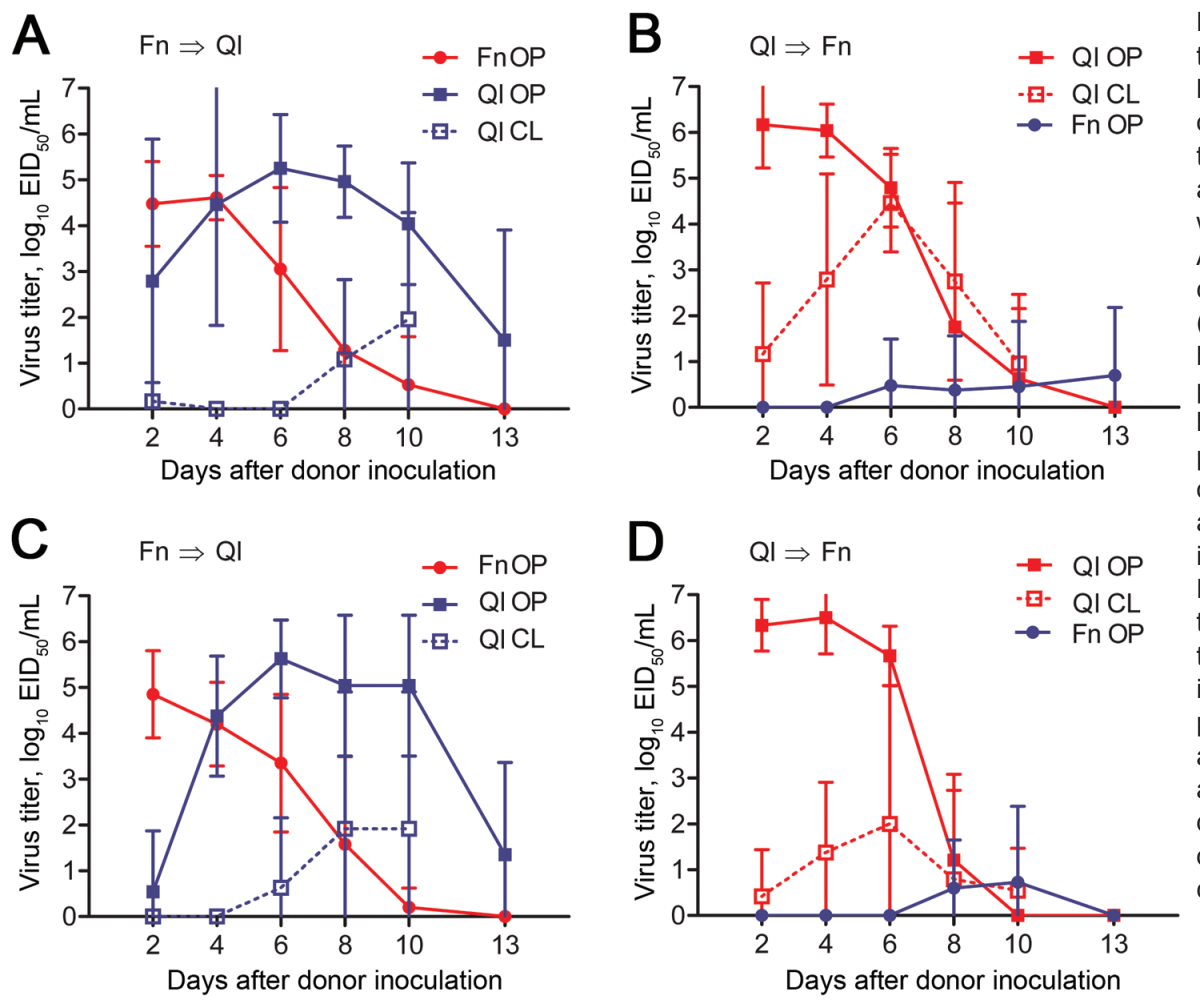
Waterborne transmission of virus between birds in an interspecies study of influenza A(H7N9) virus transmission. Finches (n = 10) and quail (n = 6) were inoculated with influenza virus strain A/Anhui/1/2013 (H7N9) (A, B) or A/chicken/Rizhao/867/2013 (H7N9) (C,D) and paired with naive birds in an environment in which physical contact was prevented but water was shared (Figure 1, panel A). Swab samples were obtained from birds every 48 h, and virus titers were determined in embryonated chicken eggs. Data are the average titer per time point ± SD. Directionality of transmission (i.e., infected→naive) is indicated in the top left of each panel. Red indicates infected animals; blue indicates naive animals. EID_50_, 50% egg infectious dose; Fn, finch; QI, quail; OP, oropharyngeal swab sample; CL cloacal swab sample.

As in the finch–chicken experiments, society finches or bobwhite quail in this experiment were inoculated with Ck/Rizhao or Anhui/1 (donors) and shared water with naive birds. As in the other experiments, all donor finches in this experiment shed both viruses an average of 10 days (Figure 3, panels A, C; Table 2). Virus was shed by the oropharyngeal route, with the exception of 1 Ck/Rizhao-inoculated finch that shed virus via the cloaca 4 dpi (3.5 log_10_ EID_50_/mL). Water-contact quail were quickly infected and shed virus by 2 dpi. 

All water-contact quail shed Anhui/1 by the oropharyngeal route (Figure 3, panels A, C; Table 2), and peak virus titers were equal to or exceeded those of the donor finches beginning at 4 dpi (AUC analysis, p = 0.0005). All water-contact quail also shed virus via the cloaca (Figure 3, panels A, C). A comparable trend was observed in Ck/Rizhao experiments: by 2 dpi, all water-contact quail shed virus by the oropharyngeal route, and cumulative titers exceeded those of the donor finches (AUC analysis, p<0.001). Only half these birds shed virus via the cloaca (Figure 3, panel C). Cumulative oropharyngeal shedding of the 2 viruses did not differ statistically in the water-contact quail.

In the converse experiment, in which quail served as donors, all quail shed Anhui/1 and Ck/Rizhao by the oropharyngeal and cloacal routes for 13 and 10 days, respectively (Figure 3, panels B, D; Table 2). Despite the high titers of virus shed from quail, transmission to the water-contact finches was infrequent. Two of 10 finches in the Anhui/1 experiment shed virus (1 shed for 7 days) (Figure 3, panel B; Table 2). Two of 10 finches in the Ck/Rizhao experiment shed virus, although only for 2 consecutive time points each (Figure 3, panel D; Table 2). Cumulative shedding of the 2 viruses did not differ statistically in the water-contact finches. Thus, when finches and quail share water resources, efficient and sustained interspecies transmission occurs from finches to quail, but only sporadic transmission occurs from quail to finches.

### Airborne Transmission between Finches and Poultry

**Figure 4:**
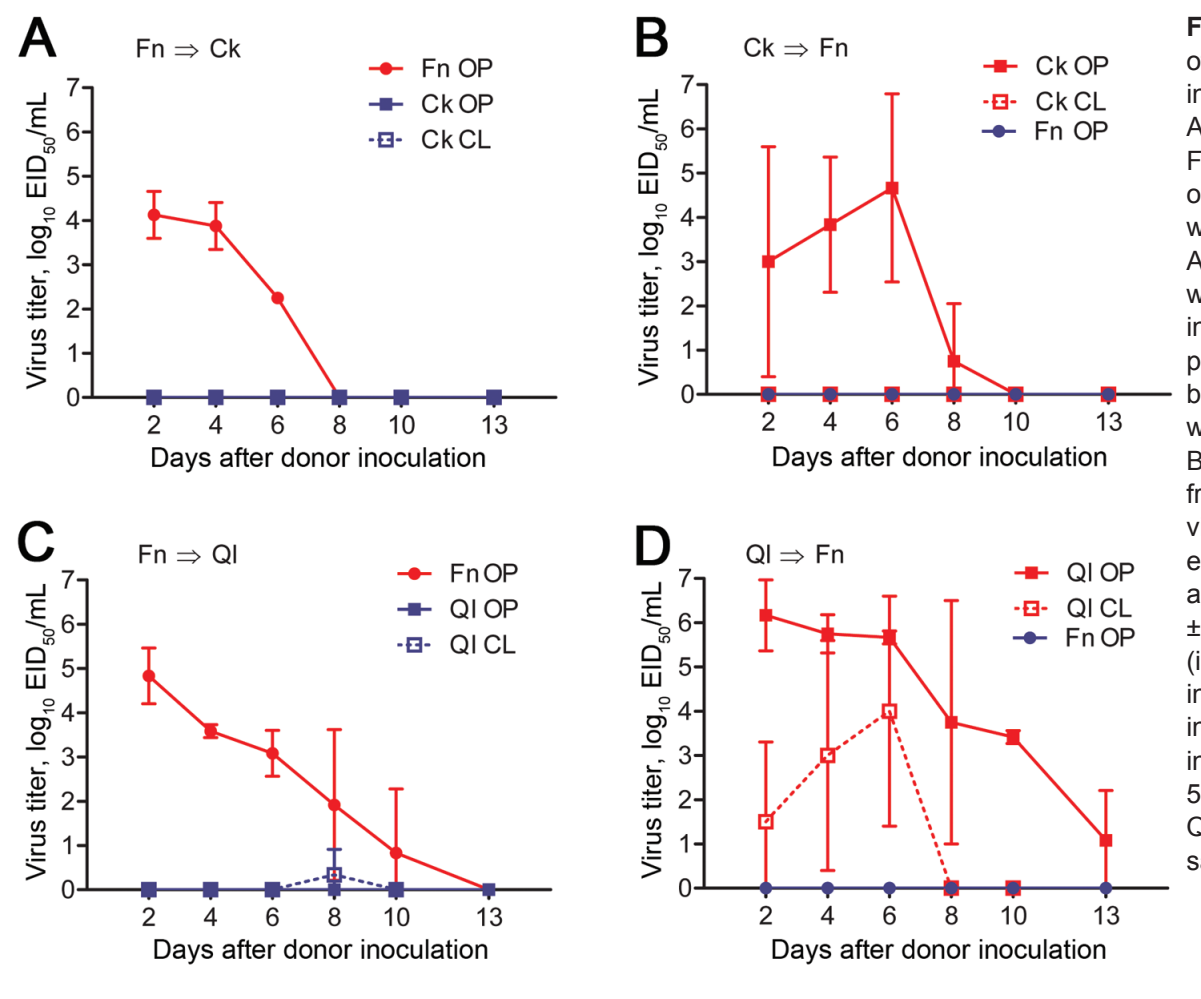
Airborne transmission of virus between birds in an interspecies study of influenza A(H7N9) virus transmission. Finches (n = 2 or 3) and chickens or quail (n = 3) were inoculated with influenza virus strain A/Anhui/1/2013 (H7N9) and paired with naive birds in an environment in which physical contact was prevented but by an air-permeable barrier and food/water resources were not shared (Figure 1, panel B). Swab samples were obtained from birds every 48 h, and virus titers were determined in embryonated chicken eggs. Data are the average titer per time point ± SD. Directionality of transmission (i.e., infected→naive) is indicated in the top left of each panel. Red indicates infected animals; blue indicates naive animals. EID_50_, 50% egg infectious dose; Fn, finch; QI, quail; OP, oropharyngeal swab sample; CL cloacal swab sample.

The birds in our experiments shared the same airspace, so we examined whether virus transmission occurred between the species via large droplet particles or smaller, fully aerosolized particles. Droplet transmission was less likely to occur because the animals were separated and droplet sources (e.g., water splashes) were minimized through placement of separate water pans. Society finches (n = 2 or 3) were housed with chickens or bobwhite quail (n = 3) (Figure 1, panel B). Consistent with birds in the previous experiments, donor finches, chickens, and quail shed virus for 6–10, 8, and 13 days, respectively (Figure 4; Table 2). Finches and chickens shed virus by the oropharyngeal route only; quail shed virus by the oropharyngeal and cloacal routes (Figure 4; Table 2). In the chicken–finch pairings, neither naive species shed virus at any time point (Figure 4, panels A, B; Table 2). In the quail–finch pairings, naive finches also shed no virus (Figure 4, panel C; Table 2), but 1 naive quail shed virus via the cloaca on dpi 8. Shedding from the naive quail was the only instance of virus detection in this group and did not meet our transmission criteria, and the shed virus was at the lower limit of detection (Figure 4, panel D). Thus, in our experimental setting, there was no airborne transmission between finches and chickens and very little if any between finches and quail.

### Virus Load in Shared Water Pans

In the previously described experiments, we observed interspecies transmission of H7N9 virus when birds shared water, but transmission did not occur when they shared airspace but not water. We hypothesized that this effect was primarily mediated by water contact. Because finches, chickens, and quail shed virus by the oropharyngeal route (often exclusively), transmission via water is possible and may occur during drinking and other events associated with water contact. To test this hypothesis, we sampled 500 μL of the water remaining in each pan on 4 different dpi and at 8 dpi (i.e., representing the water supply 4 days after a full water change).

**Figure 5:**
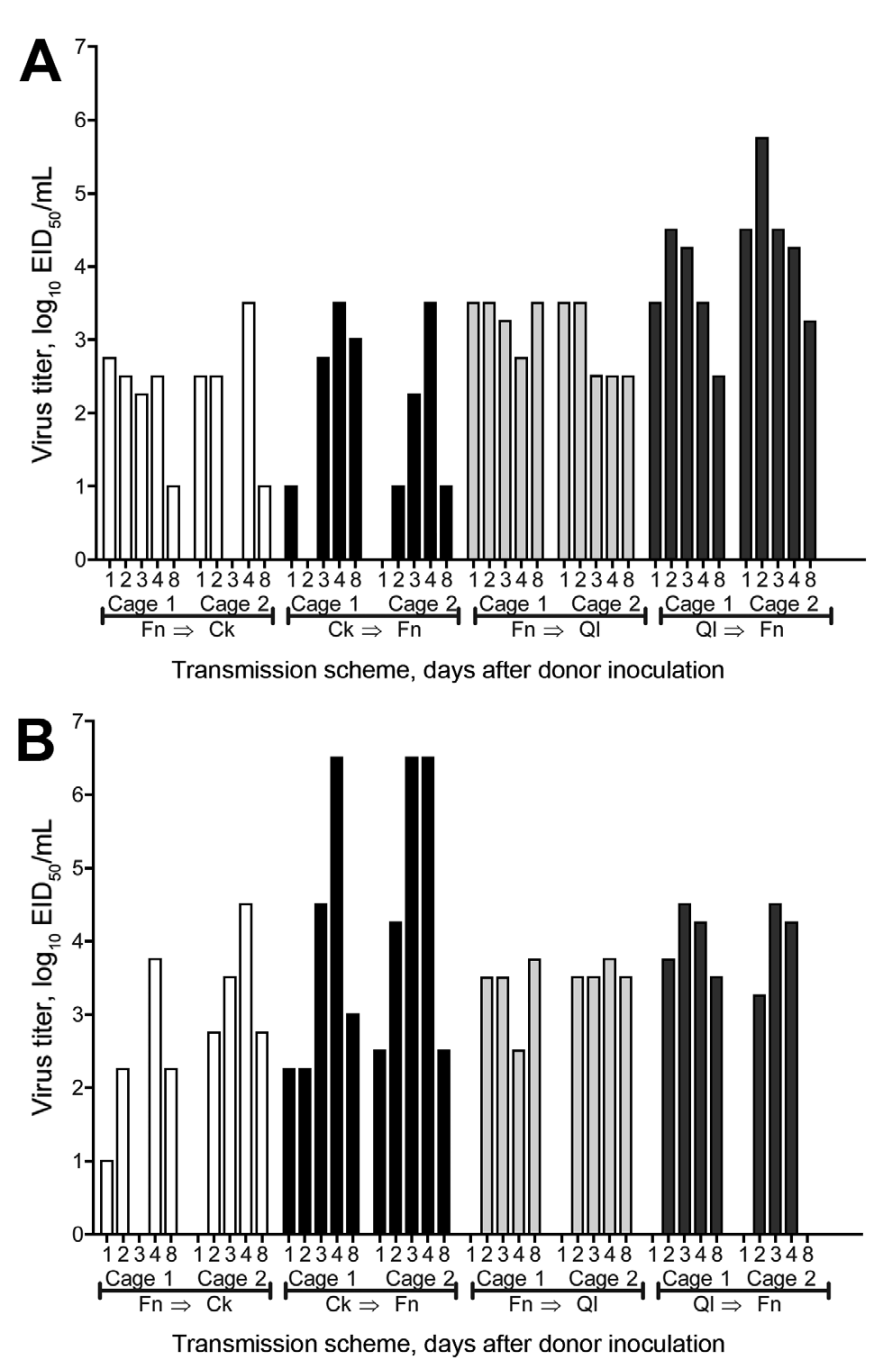
Virus load in shared water pans for birds in an interspecies study of influenza A(H7N9) virus transmission. A shared drinking water sample (500-μL) was collected daily on postinoculation days 1–4 and 8. Virus titers in samples were determined in embryonated chicken eggs. A) Shedding from A/Anhui/1/2013 (H7N9)–infected birds. B) Shedding from A/Chicken/Rizhao/867/2013 (H7N9)–infected birds. Cages 1 and 2 indicate results from duplicate experimental groups. EID_50_, 50% egg infectious dose; Ck, chicken; Fn, finch; QI, quail.

During >4 of 5 sampled time points, virus was detected in all water pans, regardless of the inoculated bird species (Figure 5). Peak virus titers in water among the 4 initial time points were 3.8–6.5 log_10_ EID_50_/mL for finch–chicken experiments and 3.8–4.5 log_10_ EID_50_/mL for finch–quail experiments (Figure 5). At 8 dpi, 4 days after a full water change, virus was still present (2.8–3.5 log_10_ EID_50_/mL) (Figure 5). Therefore, substantial and sustained amounts of infectious virus were shed from infected birds into shared water pans. Shedding patterns in our past (*13*) and present studies suggest that this virus was deposited into the water via oropharyngeal shedding. Because of variations in water levels and consumption by different species, quantitative comparisons were not possible.

### Illness among Birds and Virus Isolation from Organs

Overall, 5 of 86 finches, 4 of 30 quail, and 0 of 30 chickens died during the experiments (Tables 1, 2). All chickens remained free of clinical signs of disease, and none died. 

Two of 25 finches inoculated with Anhui/1 died at 2 and 6 dpi, respectively; neither bird had clinical signs of disease. Virus titers in trachea and lung samples were 2.3–4.5 log_10_ EID_50_/mL (Table 2).

Three contact finches died: 1/18 water contacts and 1/5 airborne contacts paired with Anhui/1-infected chickens, and 1/18 water contacts paired with Ck/Rizhao-inoculated chickens. No clinical signs of disease were observed in these birds. Virus was not isolated from swab samples for water contacts before death but was isolated from trachea and lung samples at necropsy (4.3–6.5 log_10_ EID_50_/mL) (Figure 2, panels B, D; Tables 1, 2). An airborne-contact finch that died 4 dpi showed no clinical signs, did not shed virus (Figure 4, panel B; Table 2), and had no virus in its organs (Table 2). This death was likely caused by cage stress, although the definitive cause could not be determined.

One of 9 Anhui/1-donor quail died 15 dpi; virus was isolated from its trachea (3.3 log_10_ EID_50_/mL) and lung (4.5 log_10_ EID_50_/mL) at necropsy (Table 2). Three days before death, the bird displayed hunched posture, ruffled feathers, and lethargy. All inoculated cage mates of the bird showed less severe degrees of lethargy. All Ck/Rizhao-donor quail remained free of clinical signs (Table 2).

Two of 6 naive water-contact quail in Anhui/1 experiments died at 15 dpi, and 1 of 6 naive water-contact quail in Ck/Rizhao experiments died 10 dpi (Table 1). These quail displayed clinical signs of disease (ruffled feathers, hunched posture, a drop in temperature) 2–4 days before death. Virus was detected in respiratory organs at necropsy. We noted sporadic and less severe clinical signs among contact quail in Anhui/1 experiments; these birds survived (Table 2). One water-contact quail in an Anhui/1 experiment had conjunctivitis beginning 6 dpi (data not shown), and eye swab samples were positive for H7N9 virus (4.8 log_10_ EID_50_/mL).

### Seroconversion of Finches and Poultry

Seroconversion was tested by using IDEXX ELISA. Prechallenge serum and swab samples were negative for avian influenza antibodies and virus (data not shown), strongly suggesting the lack of prior exposure to influenza A virus (Table 3).

**Table 3:** Seroconversion among birds in an interspecies study of influenza A(H7N9) virus transmission*

Challenge virus, species	No. seroconverted/no. total, by transmission route†				
	Waterborne			Airborne	
	Donor	Naive contact		Donor	Naive contact
A/Anhui/1/2013 (H7N9), donor → naive contact					
Finch → chicken	7/9	0/6		1/2	0/3
Chicken → finch	3/6	0/7		3/3	0/1
Finch → quail	6/9	4/4		2/3	0/3
Quail → finch	6/6	0/6		2/2	0/3
A/chicken/Rizhao/867/2013 (H7N9), donor → naive contact					
Finch → chicken	5/10	2/6		NT	NT
Chicken → finch	5/6	0/7		NT	NT
Finch → quail	10/10	5/5		NT	NT
Quail → finch	6/6	2/10		NT	NT
*Prechallenge serum samples from 7 finches, 5 chickens, and 5 quail were confirmed influenza virus–negative by IIDEXX ELISA. Donor birds were inoculated with the challenge virus; contacts were not inoculated. NT, not tested. †Determined by using an influenza A virus blocking ELISA (IDEXX AI MultiS-Screen Ab Test; IDEXX Laboratories, Westbrook, ME, USA). A signal-to-noise cutoff of <0.5 was considered seroconversion.					

More than half of donor birds seroconverted: for Anhui/1-inoculated birds, finches 66%–77% (range for donor groups), chickens 50%, quail 100%; for Ck/Rizhao-inoculated birds, finches 50%–100%, chickens 83%, quail 100% (Table 3). Among water-contacts, all quail seroconverted, irrespective of virus. No water-contact chickens paired with Anhui/1-donor finches seroconverted, but 2 of 6 chickens paired with Ck/Rizhao-donor finches seroconverted. Twenty percent of finches in water contact with Ck/Rizhao-donor quail seroconverted, but none of the other contact finches seroconverted. Aerosol-contact birds remained seronegative (Table 3).

## Discussion

Novel influenza A(H7N9) viruses emerged in China in 2013 and were first detected in humans with severe illness (*7*). The viruses are maintained in Chinese poultry and continue to cause human disease. We previously showed that songbirds and parakeets are susceptible to H7N9 virus and shed virus into drinking water (*13*). Here we examined interspecies transmission of H7N9 virus and demonstrated that waterborne, but not airborne, transmission occurs between society finches and poultry. Virus was more likely to transmit from chickens to naive finches than vice versa, and such transmission occurred more frequently with chicken virus (Ck/Rizhao) than human virus (Anhui/1). In contrast, virus transmitted more easily from finches to naive quail than vice versa.

H7N9 viruses and viruses with genes homologous to those of H7N9 virus have been isolated from 2 passerine birds: bramblings and a tree sparrow (*1*,*8**,**12*). We used a related passerine bird, the society finch, which originates from munias, close relatives to true finches and true sparrows. Japanese quail are prevalent in East Asia markets but were unavailable for use; thus, we used bobwhite quail (same taxonomic order/family). Bobwhite quail support influenza replication, and virus receptors in their respiratory tracts (*19*) and titers and routes of H7N9 virus shedding are similar to those for Japanese quail (*6*). We believe the model species we used can reflect the dynamic interaction and transmission events we tested.

The isolation of LPAIVs from water has been reported (*20*–*23*). Water plays a key role in the transmission of LPAIVs among waterfowl (*22*,*24*–*26*) and has experimentally been implicated in influenza virus transmission among poultry and other bird species (*27*–*29*). Therefore, our finding of waterborne transmission of H7N9 virus between finches and poultry is consistent with previous findings. However, a study by Ku et al. (*30*) demonstrated that contact transmission of H7N9 between infected and naive chickens does not occur. Presumably, the birds in that study shared a water source, but virus titers in the water were not measured, and inoculated chickens in that study shed for a shorter period than those in our study and other studies (*6*).

Airborne transmission of LPAIV among poultry has been demonstrated for multiple influenza subtypes, but Zhong et al. (*31*) reported that airborne transmission of H9N2 likely requires mutations that stabilize the HA (363K) protein, alter PA activity (672L), or both. Anhui/1 and Ck/Rizhao possess PA-672L but lack HA-363K, which may explain the lack of airborne transmission in our experiments. Our data are also in line with those of Spekreijse et al. (*32*), who reported that airborne transmission of H5N1 virus occurs at a low rate or not at all in chickens. Adaptation of H7N9 viruses in poultry or passerine species may be required for airborne transmission. For now, this route appears to represent a low risk. However, in our settings, the small number of animals used and the testing of a human-origin virus could have hindered detection of low-level airborne transmission.

We found little difference between the 2 viruses used in our study, except that replication of Ck/Rizhao was significantly better than that of Anhui/1 in donor chickens. Anhui/1 has several mutations conferring mammalian replication and receptor binding (*1*) and would be predisposed to replicate more efficiently than Ck/Rizhao in mammalian tissues. Thus, although Anhui/1 does replicate in chickens, molecular adaptions to mammals may constrain this replication. Bobwhite and Japanese quail are susceptible to a variety of influenza viruses (*33*–*36*) and have avian and mammalian influenza virus receptors in their respiratory and digestive tracts (*19*,*34*,*37*). It is not surprising that human and avian H7N9 viruses replicated to levels similar to those in these birds.

Although the type of finches used in this study would not necessarily be expected to have poultry contact, other finch and sparrow species are peridomestic and susceptible to H7N9 virus; intermingling of these birds in nonsecured poultry operations like farms or live bird markets could facilitate transmission of H7N9 virus to poultry. Chickens and, in particular, quail could then act as an amplifying host, releasing large amounts of H7N9 virus into the environment, thereby posing a health risk to humans with direct contact. Bobwhite quail and Japanese quail have been shown to be highly susceptible to LPAIVs, including subtype H7N9, and to highly pathogenic avian influenza viruses (*6*,*34*,*36*,*38*); those findings are consistent with our observations of quail as the most receptive recipient species.

We did not address interspecies transmission between birds and mammals. Passerine and psittacine birds shed H7N9 virus at levels lower than or nearly equal to those of domesticated poultry (*6**, **13*). However, data correlating virus shedding by poultry with infectivity in mammals is absent, so the correlation between levels of shed virus and transmission is not known. Nevertheless, direct transmission of H7N9 virus from passerines to humans is enhanced because of the prevalence of passerine birds as household pets. Modeling such events is difficult because of the husbandry/cohabitation of the laboratory models required (i.e., ferrets with birds). A recent study demonstrated that experimental airborne transmission of H7N9 virus from donor chickens to naive ferrets does not occur (*30*). However, for humans, the handling of contaminated water (containing virus deposited by small birds or infected poultry) should be considered a risk factor for influenza virus transmission in addition to the already identified risk factor of direct poultry contact. Root et al. (*39*) demonstrated that raccoons exposed to influenza virus–spiked water, duck eggs, or duck carcasses became infected and shed virus only when exposed to the water (*39*). In addition, subtype H7 viruses can cause conjunctivitis in mammals (*40*), so human contact with H7N9 virus–contaminated water could lead to virus inoculation by the ocular route. Cases of H7N9 virus conjunctivitis have not been reported, but incidences of conjunctivitis in poultry workers or those in contact with live poultry should be investigated and monitored.

In summary, in this follow-up of our study identifying small bird species as potential vectors of H7N9 virus (*13*), we found that waterborne transmission of human and avian H7N9 viruses occurred between society finches and poultry (chickens and bobwhite quail). Quail shed virus at the highest titers and were the most susceptible species. We conclude that finches, and likely other passerines, can act as vectors for virus transmission to poultry via shared water.
